# Sequential Application of Autologous Platelet Rich Plasma and Muscle-Derived Mesenchymal Stem Cells for Acute Tendon Injuries in Horses: Early Clinical and Ultrasonographic Outcomes in a Randomized, Double-Blind Controlled Study

**DOI:** 10.3390/ani16060940

**Published:** 2026-03-17

**Authors:** Didier Serteyn, Hélène Graide, Justine Ceusters, Maxime Vandersmissen, Alexandra Salciccia, Charlotte Sandersen, Jean-Philippe Lejeune

**Affiliations:** 1 Center for Oxygen R&D, University of Liege, Sart Tilman, 4000 Liege, Belgium; helene.graide@uliege.be (H.G.); j.ceusters@uliege.be (J.C.); charlotte.sandersen@uliege.be (C.S.); 2 Revatis SA, Aye, 6900 Marche-En-Famenne, Belgium; 3 Equine Research Center, Mont-le-Soie, 6690 Vielsalm, Belgium; jph.lejeune@montlesoie.be; 4 Equine Clinic, Faculty of Veterinary Medicine, University of Liege, Sart-Tilman, 4000 Liege, Belgium; m.vandersmissen@uliege.be (M.V.); alexandra.salciccia@uliege.be (A.S.)

**Keywords:** tendinopathy, horse, orthobiologics, mesenchymal stem cells, clinical studies

## Abstract

Tendon and ligament injuries are frequent in sport horses and are prone to recurrence due to incomplete healing. Platelet-rich plasma (PRP) and mesenchymal stem cells (MSCs) are increasingly used, but data controlled on the early effects of autologous MSCs remain limited. A prospective randomized, double-blind, placebo-controlled trial was conducted in horses with naturally occurring tendinopathies. After standardized PRP pretreatment, non-responders were randomized to receive intralesional autologous muscle-derived MSCs (mdMSCs, *n* = 17) or placebo (*n* = 6). Clinical and ultrasonographic parameters were evaluated at the recruitment period (T0) before the administration of the local treatment (T1), 4 (T2) and 8 weeks later (T3). Eighteen horses from both groups received at T2 a second intralesional injection of mdMSCs at the investigator’s discretion; this phase was not randomized. Horses treated with mdMSCs showed significant improvements from T1 to T2 across both clinical and ultrasonographic parameters, whereas no significant changes were observed in the placebo group. Between-group comparison at T2 confirmed the significant superiority of mdMSCs. Further improvements were observed between T2 and T3 in the 18 horses that received a second injection. No systemic adverse events were recorded, and local reactions were mild and transient. This randomized controlled trial demonstrates early clinical and ultrasonographic efficacy of autologous mdMSCs in equine tendinopathy, with additional benefit from a second injection. The autologous approach combines efficacy and safety while avoiding the immunological and logistical constraints of allogeneic strategies.

## Simple Summary

Tendon and ligament injuries are common in sport horses and are associated with prolonged recovery and a high risk of recurrence. Orthobiologic therapies such as platelet-rich plasma (PRP) and mesenchymal stem cells (MSCs) are increasingly used in clinical practice, but controlled data on autologous MSCs in naturally occurring lesions remain limited. In this randomized, double-blind, placebo-controlled clinical trial, horses with acute tendinopathies first received standardized leukocyte-reduced PRP. Animals that did not show sufficient clinical improvement were then randomized to receive intralesional autologous muscle-derived MSCs (mdMSCs) or placebo. To specifically assess the efficacy of mdMSCs beyond the initial PRP effect, analyses were performed in the per-protocol population excluding horses showing early clinical improvement after PRP. Horses treated with mdMSCs showed significant clinical and ultrasonographic improvement compared with placebo. A second injection in partial responders provided additional benefit. No systemic adverse effects were observed. These results support the clinical relevance of a sequential strategy combining immediate PRP treatment with delayed autologous cell therapy in equine tendon injuries.

## 1. Introduction

Tendon and ligament injuries are a leading cause of morbidity and loss of performance in sport horses, with recurrence rates often exceeding 50%, often related to the formation of mechanically inferior scar tissue during tendon healing [1]. Conventional management is largely palliative, and regenerative approaches using orthobiologics have therefore attracted major interest [2,3]. Among these, autologous platelet-rich plasma (PRP) is widely used because of its immediate availability. However, randomized controlled studies have reported inconsistent and often transient benefits, with no consistent reduction in reinjury risk. Differences in PRP preparation methods and variability in platelet and leukocyte concentrations further complicate comparisons among studies [4,5,6,7].

Mesenchymal stem cells (MSCs) represent an alternate option. Early studies suggested improved tendon architecture and reduced reinjury rates [1,8,9,10]. More recently, a multicenter randomized controlled trial demonstrated the efficacy of allogeneic PRP and tenogenic-primed MSCs at 8 and 16 weeks post injection, with reduced recurrence rates documented at 2 years [11], and a companion study confirmed their safety [12]. In addition, a systematic review and meta-analysis concluded that MSCs, alone or in combination with PRP, significantly reduce the risk of reinjury, although improvement in return to performance was not consistently demonstrated [13].

Nevertheless, the use of allogeneic MSCs entails potential immunogenicity [14,15,16,17]. This requires stringent donor selection and master cell banks, which add regulatory and logistical complexity [18]. Recently, Cequier et al. (2024) showed that MHC-mismatched MSCs, especially primed ones by a cocktail of proinflammatory cytokines or pre-differentiated in chondrocytes elicited stronger immune responses than unprimed cells [19]. In this context, MHC-mismatched refers to donor and recipient animals presenting different major histocompatibility complex (MHC) haplotypes, which may lead to immune recognition of the administered allogeneic cells. These results underscore the need for MHC matching or priming strategies to balance immunomodulation and rejection in tendon treatments.

By contrast, autologous MSCs avoid MHC mismatch and banking requirements, but their clinical application is constrained by the time required for culture and expansion. This creates a therapeutic dilemma: whether to treat immediately with an “off-the-shelf” product such as PRP or allogeneic MSCs, or to delay treatment to administer autologous MSCs.

Our approach resolves this dilemma by combining both strategies: horses received autologous PRP immediately after diagnosis, providing early biological support, followed by autologous muscle-derived MSCs (mdMSCs) once cultures were established. In addition, a subgroup received a second injection of mdMSCs four weeks later, to evaluate whether repeated dosing could consolidate tendon and ligament healing.

Skeletal muscle has been investigated as a practical source of autologous mesenchymal stem/stromal cells in horses. Muscle microbiopsy allows minimally invasive harvesting and expansion of mdMSCs, and preliminary experimental and clinical observations suggest their potential relevance for the treatment of musculoskeletal injuries

The aim of this randomized, double-blind, placebo-controlled trial was therefore to evaluate the early clinical and ultrasonographic efficacy of autologous mdMSCs administered after standardized PRP pretreatment, and to test the hypothesis that a second injection would provide additional benefit. Evaluations were scheduled at 8, 12 and 16 weeks post inclusion to capture the short-term dynamics of tendon and ligament repair.

## 2. Materials and Methods

### 2.1. Study Population

This was a multicenter, prospective, randomized, double-blind, placebo-controlled clinical trial conducted in accordance with Good Clinical Practice guidelines. This study was approved by the competent authorities of experimental animal welfare and drug registration. Five veterinary investigators were selected for this study based on their clinical background and recognized experience in equine orthopaedics. They were responsible for recruiting 30 cases from their hospital caseload based on the following inclusion criteria: Warmblood horses of various sport disciplines with acute unilateral lesion of the superficial of deep flexor tendon or the suspensory ligament, age ≥ 2 years, lesion less than 21 days old, and lameness grade 2 or 3 of the 1-in-5 AAEP scale. Lesions involved the superficial or deep digital flexor tendons and suspensory ligament branches located in the metacarpal or metatarsal regions of the limbs. Exclusion criteria included bilateral lesions, chronic injuries, or previous intralesional treatments. Owners gave informed consent to participate in this study and remained unaware of the treatment, such as the investigator was blinded to the treatment.

### 2.2. Clinical Evaluation

Lameness was graded using the AAEP lameness scale (0–5). Local pain and swelling of the superficial digital flexor tendon were assessed by palpation and scored semi-quantitatively based on tenderness and increase in tendon diameter relative to the contralateral limb (0 = none; 3 = severe). A wedge test (heel or toe elevation for 30 s followed by trotting) was performed in horses and was graded as absent (0), moderate (1), or worsening of the lameness (2). A total clinical score (TCS) (0–10) was calculated by combining lameness, pain, inflammation, and wedge test scores.

Ultrasonographic examinations were performed according to the method described by Rantanen et al. [20] using a 5–7.5 MHz linear transducer, with transverse and longitudinal scans. Digital images were stored for analysis. The maximal injury zone (MIZ) was identified, and lesions were classified as core, marginal, or diffuse. Total cross-sectional area (T-CSA) and lesion cross-sectional area (TL-CSA) were measured, and lesion size was expressed as a percentage of T-CSA. Echogenicity was graded on a four-point scale (0 = normoechoic; 3 = anechoic). Fiber alignment score (FAS) was assessed on longitudinal images based on the estimated proportion of parallel fibers within the lesion (0 = >75%; 3 = <25%). Lesions involved the superficial digital flexor tendon, deep digital flexor tendon, or suspensory ligament branches. Ultrasonographic evaluation included assessment of lesion localization, fiber alignment score (FAS), echogenicity, and percentage of lesion area relative to the tendon cross-sectional area. In order to minimize operator-related variability, each horse was examined throughout the study by the same investigator.

### 2.3. Study Design

At inclusion (T0), all recruited horses received an intralesional injection of autologous leukocyte-reduced PRP, and a muscle microbiopsy was performed simultaneously for preparation of autologous mdMSCs.

After expansion of the cells, horses that did not show sufficient clinical improvement—defined as a decrease of less than three points in the total clinical score—were randomized at T1 to receive either intralesional autologous mdMSCs (two-thirds) or placebo (one-third).

According to the study protocol presented to investigators and horse owners at inclusion, the treatment sequence included a second intralesional administration of mdMSCs four weeks later (T2). In practice, this second injection was performed in most horses. However, in a limited number of cases showing rapid and marked clinical improvement, investigators decided not to perform the second administration. These decisions were taken while the study remained blinded and without knowledge of treatment allocation.

Final clinical and ultrasonographic evaluations were performed four weeks after T2 (T3), following the same standardized protocol.

### 2.4. Preparation of PRP

Autologous leukocyte-reduced PRP was prepared using the Axovet BMC/PRP kit (Revatis SA, Aye, Belgium) according to the manufacturer’s protocol. Two tubes of 8 mL venous blood were collected aseptically from the jugular vein into dedicated PRP tubes. After centrifugation for 8 min at 1500× *g*, neutrophils and erythrocytes were effectively eliminated. Under sterile conditions, platelet-poor plasma was removed, and around 2 mL of PRP containing 300–400 · 10^3^ platelets by µL with a WBC count less than 1 · 10^3^/µL was recovered per tube following gentle resuspension. PRP was injected intralesionally immediately after preparation.

### 2.5. Muscle Microbiopsy and Cell Culture

At T0, a muscle microbiopsy was performed on the triceps brachii under sedation and local anesthesia. One milliliter of mepivacaine (Intra-Epicaine^®^, Dechra, Herentals, Belgium) was injected subcutaneously before sampling. Approximately 20 mg of muscle tissue was collected using a microbiopsy needle (14G) and placed in DF-12 transport medium [DMEM/Ham’s F12 with HEPES and glutamine, 5 mL penicillin (1000 U/mL)–streptomycin (10,000 µg/mL), and 2.5 mL amphotericin B (250 µg/mL)]. Samples were stored at 4 °C for a maximum of 72 h before culture initiation.

In the laboratory, adherent cells were expanded under GMP conditions. After 6 to 8 weeks, 4 to 6 · 10^7^ autologous mdMSCs were obtained for clinical use. Quality control included sterility, endotoxin and mycoplasma testing, viability assessment, and phenotypic characterization consistent with mesenchymal stromal cells [21].

Identity and purity of the finished product are assessed by flow cytometry performed on frozen and thawed cell suspension samples. Identity is confirmed by demonstrating expression of the mesenchymal stem cell markers CD44 and CD90 at levels equal to or greater than 70%. Purity is established by confirming minimal expression (≤2%) of hematopoietic and immunogenic markers CD45 and HLA-DR. Post-thaw viability is assessed using the validated Trypan Blue exclusion method (Ph. Eur. 2.7.29). Acceptance criteria require a viability of at least 75% immediately after thawing.

### 2.6. Treatment Administration

Horses randomized to the treatment group received 10^7^ autologous mdMSCs suspended in 1 mL Cryostor CS5^®^, while placebo horses received 1 mL Cryostor CS5^®^ alone. Treatment or placebo were thawed immediately before injection. Randomisation allocated 20 horses to the treatment group and 10 horses to the placebo group. Injections were performed under sedation and loco-regional anesthesia, guided by ultrasound into the lesion.

A protective bandage was applied for 10 days, followed by a gradual return to controlled exercise as tolerated.

After treatment, a controlled rehabilitation program was recommended, consisting of an initial period of restricted activity followed by progressive hand walking and gradual reintroduction of exercise according to clinical evolution. Because this was a clinical study involving client-owned horses, rehabilitation was adapted by the attending clinician and monitored during follow-up examinations.

Safety was monitored by recording all local and systemic adverse events.

### 2.7. Statistical Analysis

Within-group changes were analyzed using the Wilcoxon signed-rank test; between-group comparisons were performed using the Mann–Whitney U test; and longitudinal effects across multiple time points were assessed using Friedman’s test. Analyses were conducted on the per-protocol population, with exclusion of PRP responders to avoid early confounding effects. Complete or near-complete recovery was defined as a Total Clinical Score (TCS) of 0–1 at T3.

## 3. Results

The investigators determined the diagnosis of acute unilateral tendinopathy of the superficial (SDFT, *n* = 12), deep digital flexor tendon (DDFT, *n* = 5) or a desmopathy of the suspensory ligament (*n* = 13) based on specific examinations. Thirty-four sport horses with acute unilateral lesions of the superficial or deep flexor tendon or the suspensory ligament were initially recruited, but 4 horses dropped out before the injection of the mdMSCs or placebo for reasons unrelated to the study. Thirty horses completed the study: 17 males (gelding) and 13 females with a mean aged of 13 ± 5 years.

### 3.1. Overall Efficacy (Inclusive Population, T0 → T3, N = 30)

Longitudinal analysis of median values and interquartile range (IQR) are reported in Table 1 and showed a significant effect of time for all evaluated clinical and ultrasonographic parameters. Two horses were not included in the T0–T3 analysis due to early clinical recovery and discontinuation of follow-up after T2. Median Total Clinical Score decreased from 6 at T0 to 1 at T3. Similar reductions were observed for AAEP lameness score, wedge test response, fiber alignment score, echogenicity score, and lesion size. Friedman tests demonstrated a significant longitudinal effect for all parameters (all *p* < 0.0001), and paired Wilcoxon analyses confirmed significant differences between baseline and final follow-up (T0 vs. T3; all *p* < 0.0001).

### 3.2. Specific Efficacy of mdMSCs (Non-PRP Population, T1 → T2)

To specifically evaluate the therapeutic effect of mdMSCs beyond PRP, 7/30 horses that responded positively to the initial PRP injection were excluded from this analysis. This non-PRP population comprised 17 horses treated with mdMSCs and 6 receiving placebo. At inclusion, lesion severity was characterized using ultrasonographic parameters including fiber alignment score (FAS), echogenicity, and percentage of lesion area relative to the tendon cross-sectional area. Baseline ultrasonographic findings and lesion distribution were comparable between the treatment groups.

Between T1 and T2, horses treated with mdMSCs showed significant improvement in all main clinical and ultrasonographic parameters. TCS decreased markedly in the mdMSC group (6.0 → 4.0; *p* = 0.0005), while it remained essentially unchanged in the placebo group (6.0 → 5.0; *p* > 0.05). Similar observations between T2 versus T1 were observed for AAEP grade (*p* = 0.001), Wedge (*p* = 0.012) FAS (*p* = 0.020), echogenicity (*p* = 0.0584), and lesion size, confirming a consistent benefit of mdMSC therapy, whereas no significant variations were observed in the placebo group between T1 and T2.

**Figure 1:**
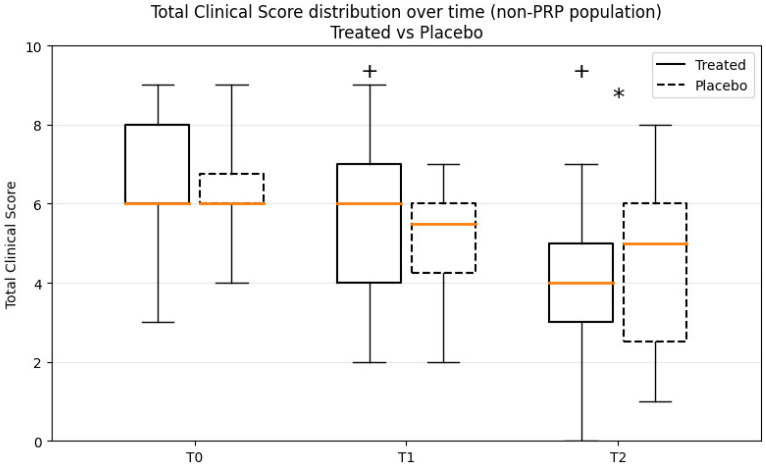
Box-plot representation of Total Clinical Score (TCS) at baseline (T0), 8 weeks (T1) and 12 weeks (T2) in treated and placebo horses, excluding PRP responders. Boxes represent interquartile ranges, horizontal lines indicate medians, and whiskers indicate minimum and maximum values. Lower scores indicate clinical improvement. + indicates a significant within-group improvement between T1 and T2 in treated horses (Wilcoxon signed-rank test, *p* < 0.001). * indicates a significant treatment effect at T2 compared with placebo (Mann–Whitney U test on Δ(T2–T1), *p* = 0.018).

Between-group comparisons demonstrated the superiority of mdMSCs over placebo, with a significant difference for TCS (*p* = 0.017). These results are shown in Figure 1.

### 3.3. Second Injection Effect (T2 → T3, N = 18)

Of the 30 horses included in the study, 24 received at T2 an intralesional injection of mdMSCs at the investigator’s discretion Among the PRP responders, the second injection was not associated with further measurable clinical improvement, likely due to already very low clinical scores prior to re-injection.

In contrast, among PRP non-responders, a second injection was administered in 5/6 horses in the placebo group and 13/17 in the treated group. The single placebo horse not re-injected was withdrawn between T1 and T2 due to recurrence and owner decision. When considering these 18 non PRP-responding horses as a combined population, significant changes were observed across several clinical and ultrasonographic parameters between T2 and T3.

The median TCS decreased from 4.0 to 2.5 (*p* = 0.001), while the AAEP lameness grade improved from 2.0 to 1.0 (*p* = 0.013). Ultrasonographic parameters also showed consistent benefits, with FAS increasing from 2.0 to 1.0 (*p* = 0.008) and lesion size reducing from 30% to 20.6% (*p* = 0.008). The wedge test improved from 1.0 to 0.0 (*p* = 0.005), indicating enhanced functional recovery. Echogenicity displayed a favorable but non-significant trend (*p* = 0.059).

These results confirm that a second injection of mdMSCs provided additional clinical and structural benefits in partial responders, consolidating tendon and ligament healing beyond the effect of a single administration.

### 3.4. Safety

No systemic adverse events were reported. Local reactions (transient swelling or pain at the injection site) were mild and resolved spontaneously. One horse received an IV injection of 1 g enylbutazone for 3 days.

## 4. Discussion

This multicenter randomized, double-blind, placebo-controlled trial shows that autologous mdMSCs, given after standardized leukocyte-reduced PRP, confer a specific and clinically meaningful benefit in horses with acute tendinopathies and desmopathies. In animals that did not show sufficient early improvement after PRP treatment, mdMSCs treatment resulted in significant improvements in lameness, composite clinical score and ultrasonographic parameters between T1 and T2. The primary outcome of the study was achieved, as a statistically significant treatment-related improvement was demonstrated for the TCS. In contrast, early between-group comparisons did not reach statistical significance for all secondary clinical and ultrasonographic parameters, which can be explained by the limited discriminative resolution of ordinal scoring systems, based on a small number of categorical grades, and by the short time interval between evaluations, particularly between T1 and T2.

In addition, analyses focusing on horses receiving a second mdMSCs injection demonstrated significant improvements across several secondary parameters between T2 and T3, supporting the presence of an ongoing and biologically coherent healing process. These findings indicate that tendon regeneration is progressive and cumulative over time, and that the second administration may be consistent with ongoing tissue remodeling and consolidation of the healing process. However, this interpretation should be considered with caution given the absence of a non-reinjected comparison group and the limited sample size.

Rehabilitation is a key factor influencing tendon healing. Although a controlled exercise program was recommended for all horses, rehabilitation protocols were adapted according to clinical evolution and management conditions of client-owned horses. Consequently, complete standardization of exercise intensity and progression across all cases could not be ensured and may have contributed to some variability in outcomes.

Previous controlled and experimental studies on PRP alone have reported inconsistent and often transient benefits, with no robust or consistent reduction in reinjury rate and substantial heterogeneity related to preparation methods and leukocyte/platelet content. A recent systematic review confirmed that PRP cannot yet be considered a standalone, reliably disease-modifying therapy for equine tendon and ligament injuries [7]. Biologically, leukocyte-reduced PRP may prime the lesion through growth factor delivery and reduced inflammation, creating favorable conditions for subsequent MSC engraftment and paracrine activity [21]. Accordingly, all horses received a standardized leukocyte-reduced PRP as an initial biologic intervention at diagnosis. This design allowed the study to control for PRP exposure while focusing the randomized comparison on those horses that failed to reach a predefined threshold of clinical improvement. The absence of between-group differences after PRP alone (T1), followed by a significant divergence between mdMSC and placebo groups at T2, indicates that the benefits observed in the randomized phase can be attributed to the mdMSCs.

One of the main challenges in regenerative medicine lies in the compromise between the need for rapid intervention and the time required to produce autologous cells [18]. Our protocol proposes a sequential approach: PRP as an immediate first-line intervention, followed by autologous mdMSCs, which ensures adequate treatment without compromising the benefits of autologous therapy.

Our findings are concordant with recent comprehensive reviews that have synthesized clinical and experimental evidence supporting MSC-based therapies for equine tendon core lesions, including improved return to performance and reduced reinjury rates [10,22,23]. Earlier clinical and experimental work with bone-marrow- and adipose-derived MSCs reported improved tendon architecture, better ultrasonographic scores and lower recurrence rates compared with conventional management alone [8,9]. A multicenter randomized trial with a combined product of tenogenic-primed allogeneic MSCs and allogenic PRP demonstrated significant improvements at 8 and 16 weeks and a reduced reinjury rate at 2 years [11]. In addition, a systematic review and meta-analysis concluded that MSCs, alone or combined with biologics such as PRP, significantly reduce the risk of reinjury even if gains in return to performance are more variable [13]. Within this context, the present study is one of the few randomized, double-blind, placebo-controlled trials evaluating an autologous mdMSC product in naturally occurring lesions, thereby adding high-quality, practice-relevant evidence to the field.

The age range of the horses included in this study was relatively broad and reflects the population commonly encountered in clinical practice. Although age-related differences in tissue healing capacity may influence treatment response, the present study was not powered to perform subgroup analyses according to age or training level.

An important strength of this work is the use of autologous mdMSCs obtained via a minimally invasive muscle microbiopsy, expanded under controlled conditions and administered at a high viable cell dose [20]. This approach is particularly attractive given the increasing recognition that MSCs are not immune-privileged and that allogeneic products can elicit both humoral and cellular immune responses in horses [14,15,17].

Reports of alloantibody formation and complement-mediated cytotoxicity against allogeneic MSCs, along with stronger immune responses to MHC-mismatched and primed cells, highlight the potential advantages of autologous strategies in terms of immunological safety [16,19]. Autologous mdMSCs may offer a potential immunological advantage compared with allogeneic cell therapies, as suggested by previous studies reporting immune recognition of MHC-mismatched MSCs in horses. However, immune responses were not specifically assessed in the present study, and this aspect should therefore be considered as a theoretical advantage supported by external literature rather than a finding directly demonstrated here.

Our data show that despite the production delay, autologous cells can be integrated into clinical workflows when combined with an initial PRP injection, thus reconciling efficacy with practicality. Because all horses initially received PRP as part of the standardized protocol and the study was conducted under double-blind conditions, early clinical improvement attributable to PRP could only be identified after completion of the study and unblinding. In a subset of horses, lesion regression after PRP alone was sufficiently marked that further evaluation of a potential treatment effect became difficult. This was taken into account in the interpretation of the comparative analyses.

The 2:1 randomization ratio, favoring mdMSCs over placebo, was chosen deliberately in a population of client-owned sport horses to facilitate owner acceptance and recruitment while preserving a concurrent placebo group and full blinding. Although this design led to a relatively small placebo arm, the study still demonstrated consistent and statistically significant differences in key clinical and ultrasonographic endpoints between mdMSC and placebo groups, supporting that the trial had sufficient power to detect clinically relevant effects. Further, the decision to administer a second injection at T2 was based on the investigator’s clinical judgment rather than on predefined objective criteria for partial response. Because neither the owners nor the investigators were aware of the treatment allocation at that time, it is possible that a second injection was requested or administered to ensure that the horse received an active treatment, even in cases where sufficient clinical improvement had already been observed. Rehabilitation and return-to-work protocols were not fully standardized. Furthermore, different lesion locations (SDFT, DDFT, suspensory ligament) were analyzed together, and the follow-up was limited to 8 weeks for the primary analyses, precluding direct assessment of long-term reinjury and performance outcomes.

By demonstrating early and specific efficacy, our findings support autologous mdMSCs as a safe and effective option for equine tendon and ligament injuries. The sequential strategy used here reflects real-world decision making: an immediately available biologic treatment at diagnosis, followed by targeted cell therapy in insufficient responders. In addition, the minimally invasive microbiopsy technique opens the possibility of preventive autologous cell banking in high-performance horses, making mdMSCs potentially available “off the shelf” in case of injury and further increasing the practicality of this approach. Overall, this trial provides robust evidence that autologous mdMSCs can be integrated into equine practice as a valuable addition to current therapeutic strategies for tendinopathy, with early structural and clinical benefits and an excellent tolerance profile. Beyond equine medicine, this paradigm illustrates how combining an immediately available biological product with patient-specific MSCs can overcome practical barriers to autologous therapies.

## 5. Conclusions

This randomized, double-blind, placebo-controlled trial demonstrates that autologous muscle-derived mesenchymal stem cells provide a significant early clinical and ultrasonographic benefit in horses with acute tendon and ligament injuries that do not sufficiently respond to initial PRP treatment. The observed improvements were consistent across clinical scores and imaging parameters and were further reinforced after a second injection in partial responders.

The sequential approach used in this study offers a pragmatic framework that reconciles the need for early intervention with the production time required for autologous cells. Within the limits of the study design and follow-up duration, autologous mdMSCs appear to represent a safe and clinically relevant option for equine tendinopathy management.

## Figures and Tables

**Table 1:** Longitudinal evolution of clinical and ultrasonographic parameters (T0–T3).

Parameter	T0 Median (IQR)	T1 Median (IQR)	T2 Median (IQR)	T3 Median (IQR)	Statistical Analysis
**Total Clinical Score (TCS)**	6 (5–7)	6 (5–7)	3 (2–5)	1 (0–2)	Friedman *p* < 0.0001 Wilcoxon T0–T3 *p* < 0.0001
**AAEP lameness score**	2 (2–3)	2 (2–3)	2 (1–2)	1 (0–1)	Friedman *p* < 0.0001 Wilcoxon T0–T3 *p* < 0.0001
**Wedge test**	2 (1–2)	2 (1–2)	1 (1–2)	0 (0–1)	Friedman *p* < 0.0001 Wilcoxon T0–T3 *p* < 0.0001
**Fiber Alignment Score (FAS)**	2 (2–3)	2 (2–3)	2 (1–2)	1 (0–1)	Friedman *p* < 0.0001 Wilcoxon T0–T3 *p* < 0.0001
**Echogenicity score**	2 (2–3)	2 (2–3)	2 (1–2)	1 (0–1)	Friedman *p* < 0.0001 Wilcoxon T0–T3 *p* < 0.0001
**Lesion size (% TL-CSA)**	38 (28–52)	36 (26–48)	28 (18–40)	15 (8–22)	Friedman *p* < 0.0001 Wilcoxon T0–T3 *p* < 0.0001

## Data Availability

The data generated in this study are part of a regulatory submission to the European Medicines Agency (EMA) conducted under the supervision of the Belgian Federal Agency for Medicines and Health Products (FAMHP/AFMPS) (authorization numbers 0003050, 0004780, 0006446, 0006764, 0007869). Due to regulatory and confidentiality constraints related to the marketing authorization process, the data are not publicly available.
